# Endothelial cells promote 3D invasion of GBM by IL-8-dependent induction of cancer stem cell properties

**DOI:** 10.1038/s41598-019-45535-y

**Published:** 2019-06-21

**Authors:** Michael G. McCoy, Dennis Nyanyo, Carol K. Hung, Julian Palacios Goerger, Warren R. Zipfel, Rebecca M. Williams, Nozomi Nishimura, Claudia Fischbach

**Affiliations:** 1 Nancy E. and Peter C. Meinig School of Biomedical Engineering, Cornell University, Ithaca, NY 14853 United States; 2 Kavli Institute at Cornell for Nanoscale Science, Cornell University, Ithaca, NY 14853 United States

**Keywords:** Biomaterials - cells, Tissues, Extracellular matrix, Cancer stem cells

## Abstract

Rapid growth and perivascular invasion are hallmarks of glioblastoma (GBM) that have been attributed to the presence of cancer stem-like cells (CSCs) and their association with the perivascular niche. However, the mechanisms by which the perivascular niche regulates GBM invasion and CSCs remain poorly understood due in part to a lack of relevant model systems. To simulate perivascular niche conditions and analyze consequential changes of GBM growth and invasion, patient-derived GBM spheroids were co-cultured with brain endothelial cells (ECs) in microfabricated collagen gels. Integrating these systems with 3D imaging and biochemical assays revealed that ECs increase GBM invasiveness and growth through interleukin-8 (IL-8)-mediated enrichment of CSCs. Blockade of IL-8 inhibited these effects in GBM-EC co-cultures, while IL-8 supplementation increased CSC-mediated growth and invasion in GBM-monocultures. Experiments in mice confirmed that ECs and IL-8 stimulate intracranial tumor growth and invasion *in vivo*. Collectively, perivascular niche conditions promote GBM growth and invasion by increasing CSC frequency, and IL-8 may be explored clinically to inhibit these interactions.

## Introduction

Glioblastoma (GBM) is defined as a high grade astrocytoma and represents the most common primary malignant brain tumor in adults with a median survival time of 15 months^1^. The poor clinical outcome of GBM patients is commonly attributed to two phenomena: (*i*) the highly invasive nature of GBM that makes complete surgical removal impossible^2^ and (*ii*) the presence of cancer stem cells (CSCs)^3^. CSCs constitute a subpopulation of GBM tumor cells that share characteristics of stem cells including self-renewal and multi-lineage differentiation but are additionally capable of tumor formation and mediating therapy resistance^4^. Importantly, CSCs are highly migratory, and increasing experimental evidence suggests that the invasive nature of GBM is related to the intracranial spreading of CSCs^5^. Nevertheless, the mechanisms that increase stem-like characteristics in GBM tumor cells and their related effects on invasion are not well characterized.

As GBM tumor cells and CSCs invade into the brain parenchyma, they engage with physical migration paths along white matter tracts and blood vessels^6^. GBM invasion along the vasculature is of particular importance because increased blood vessel density is a hallmark of GBM^7^ and thus, may promote tumor spread by simply increasing the quantity of paths that are available to tumor cells as they invade. Moreover, the physical association of CSCs with blood vessels in perivascular niches is critical to CSC self-renewal and maintenance and therefore, could support invasion by promoting putative CSC characteristics^8^. Interactions between endothelial cells and CSCs are bi- rather than unidirectional as CSCs can stimulate the formation of new blood vessels due to their proangiogenic capabilities^9,10^. Hence, GBM invasion, vascularization, and CSCs are closely intertwined. However, the molecular and cellular mechanisms that regulate GBM invasion as a function of CSC-perivascular niche interactions remain poorly understood due in part to a lack of model systems that allow studying 3D cellular invasion of patient-derived GBM tumor cells in the presence of human brain endothelial cells.

While extracellular matrix (ECM) in the brain parenchyma primarily consists of basement membrane and proteoglycan- and hyaluronic acid-rich matrix^11^, the perivascular tissue in which CSCs reside and migrate is enriched for collagen type I^11,12^. Collagen, in turn, can regulate tumor cell invasion by directing cell migration via structural and mechanical cues^13,14^, a process that is also exploited by CSCs^15^ and is likely accentuated by endothelial cell-secreted factors^16^. Hence, studying GBM invasion in a collagen type I-based model that mimics multicellular interactions between GBM tumor cells, CSCs, and endothelial cells may help reveal mechanisms contributing to GBM invasion within the perivascular space.

While many different signaling molecules have been suggested as regulators of CSCs, interleukin-8 (IL-8) may be particularly important given its roles in directing angiogenesis, invasion, and CSC behavior^17,18^. More specifically, IL-8 can promote vascular sprouting by modulating growth factor sensitivity and mobilizing matrix remodeling enzymes^19,20^. Furthermore, IL-8 upregulates stem cell marker expression in GBM and other cancers^21^ and activates various signaling pathways associated with tumorigenesis including Signal Transducer and Activator of Transcription 3 (STAT3), phosphoinositide 3-kinase (PI3K), and Mitogen Activated Protein Kinase (MAPK)^21–23^. Studying the role of IL-8-dependent changes of tumor cell invasion *in vitro*, however, is subject to significant limitations and requires relevant 3D models as (*i*) cellular IL-8 expression/secretion is significantly reduced in monolayer culture relative to *in vivo* scenarios^24^ and (*ii*) tumor cells respond to IL-8 with increased migration/invasion in 3D, but not in 2D cultures^25^.

Here, we utilized a collagen-based, microfabricated hydrogel platform to investigate the role of 3D heterotypic interactions between patient-derived GBM tumor cells and endothelial cells and determined their impact on GBM growth and invasion. Furthermore, we examined the role of IL-8 in this process and validated the potential functional significance of our *in vitro* results *in vivo*. Our data underline the importance of 3D model systems in studying GBM-microenvironment interactions as a function of IL-8 signaling and help improve understanding of the molecular mechanisms regulating GBM invasion.

## Results

### Invasion of collagen-embedded GBM spheroids correlates with CSC characteristics

**Figure 1:**
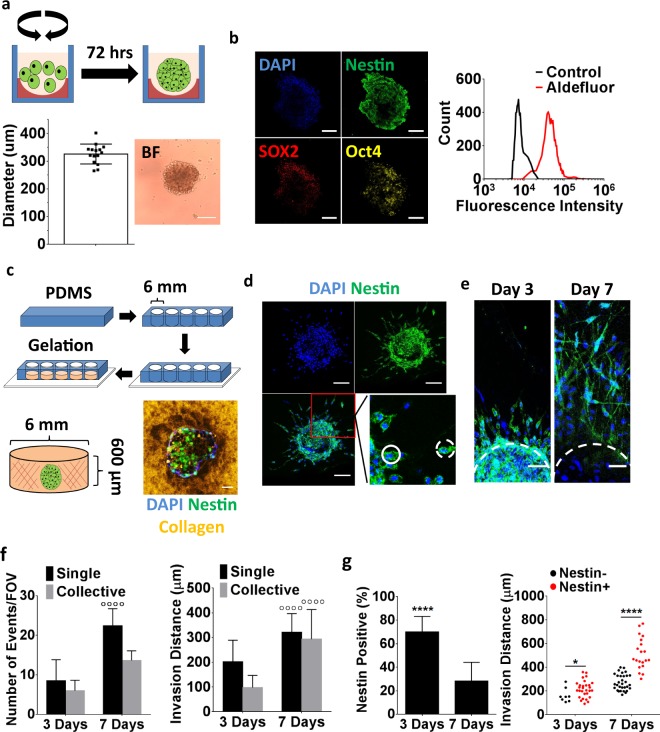
Analysis of Glioblastoma (GBM) invasion using collagen-embedded GBM spheroids. (**a**) Schematic of GBM spheroid formation. Patient-derived GBM cells (green) were seeded into agarose (red)-coated plates and allowed to form spheroids during dynamic culture on an orbital shaker. Analysis of bright field images showing uniform spheroid sizes. (**b**) Cyrosectioning and immunofluorescent staining of GBM spheroids for the stem cell markers nestin, Oct4, and SOX2. Flow cytometric analysis of GBM spheroids for the stem cell marker aldehyde dehydrogenase using the Aldefluor^TM^ assay; shown relative to the assay control. Scale bars are 100 μm. (**c**) Schematic depicting the embedding of GBM spheroids into collagen-filled poly(dimethylsiloxane) (PDMS) microwells that were sealed onto a glass coverslip for imaging purposes. Confocal micrograph of a collagen-embedded, immunostained GBM spheroid. Collagen was imaged in reflectance mode. Scale bar is 50 μm. (**d**) Confocal images of immunostained spheroids 3 days after embedding showing individual (dashed circle) and collective (solid circle) invasions of nestin-positive tumor cells. Scale bars are 100 μm. (**e**) Confocal micrographs indicating tumor cell invasion after 3 and 7 days of collagen culture. Scale bars are 50 μm. (**f**) Confocal image analysis of invasion frequency and distance. ^◦◦◦◦ ^Indicates P < 0.0001 relative to day 3 of the same condition. (**g**) Confocal image analysis of nestin-positive cells and their respective invasion distance over time. * and **** Indicate P-values < 0.05 and 0.0001, respectively.

Primary GBM spheroids are frequently used to study glioma tumorigenesis *in vitro* as this approach preserves the genomic profile of a patient’s tumor more reliably than culture in 2D monolayers^26^. GBM spheroids are typically generated by expanding primary tumor cells in non-adherent culture flasks under serum-free culture conditions. This approach yields cellular aggregates that exhibit physiologically relevant 3D cell-cell and cell-ECM interactions as well as oxygen and soluble factor gradients that not only contribute to preserving genetic stability, but also lead to enrichment of CSCs^27^. Nevertheless, the sizes of the spheroids formed by this approach can vary widely potentially impacting the number of CSCs and thus, analysis of invasion responses. To circumvent these limitations, we generated GBM spheroids of uniform size distribution by plating GBM tumor cells into agarose-coated 96-wells under serum-free culture conditions (Fig. 1a). In contrast to conventional spheroid formation protocols, this approach generated uniformly sized spheroids with an average diameter of 325.8 +/− 35.93 μm (Fig. 1a). This size is below the oxygen diffusion limit and thus, yields spheroids without central necrosis. Immunostaining of cryosections against the stem cell markers nestin, SOX2 and Oct4 suggested that the spheroids contained a population of stem-like tumor cells. We have previously confirmed that patient-derived GBM cells isolated and cultured under similar media conditions and expressing nestin, SOX2, and Oct4 can differentiate into different neural lineages^18^. Additionally, quantification of aldehyde dehydrogenase (AlDh) activity via the Aldefluor® assay, another indicator of stemness^28^, confirmed that the majority of cells in the spheroids expressed a stem-like phenotype (Fig. 1b). As nestin staining reliably correlated with all other assessed markers of stemness in these studies, it was used in the following experiments as an indicator of stemness.

To investigate invasion of GBM spheroids into ECM that may be present in the perivascular niche, spheroids were embedded into type-1 collagen hydrogels (Fig. 1c). Confocal reflectance analysis immediately following embedding of the spheroids confirmed that nestin-positive cells were in direct physical contact with collagen (Fig. 1c). After 3 days in culture, both nestin positive and negative tumor cells had invaded the hydrogel using single cell and collective cell migration modes, an observation that was even more pronounced after 7 days (Fig. 1d,e). Although GBM cells invaded more frequently in the form of single cells rather than collectively, the invasion distance in both scenarios was comparable (Fig. 1f). This is consistent with previous observations that tumor cells exhibit bi-modal forms of invasion^29^. While the number of nestin positive cells decreased upon embedding of spheroids into collagen (Fig. 1g, left), nestin positive cells constituted the majority of invasions on day 3 and had invaded over longer distances by day 7 (Fig. 1g, right). These results suggest a direct link between GBM invasion and nestin positivity in the presented *in vitro* model, thus providing a platform to mimic previously described *in vivo* scenarios in which stem-like tumor cells contributed to GBM invasion^30^.

### Brain endothelial cells stimulate CSC invasion and stem cell marker expression

**Figure 2:**
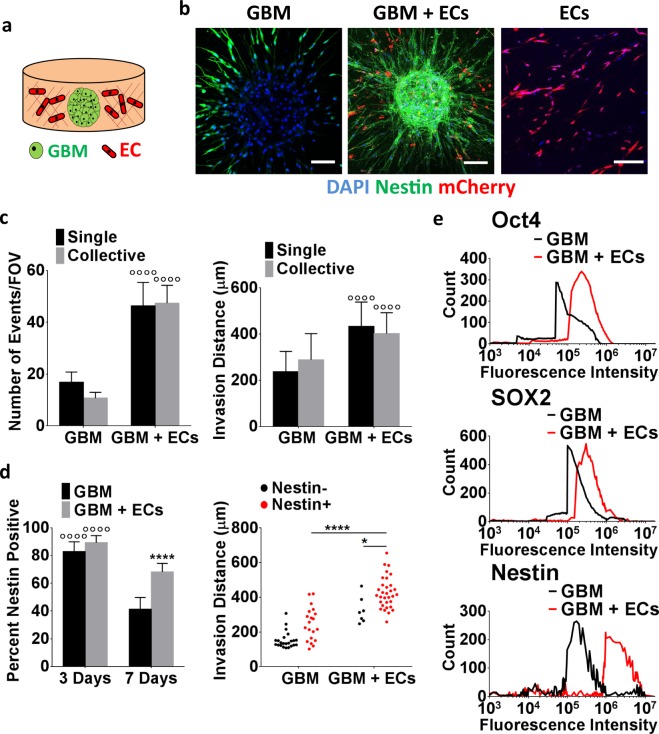
3D co-culture with brain endothelial cells promotes invasion of GBM spheroids. (**a**) Schematic of the collagen-embedded 3D co-culture model. (**b**) Confocal micrographs visualizing invasion of immunostained GBM monocultures, monocultures of mCherry-labeled brain endothelial cells (ECs), and co-cultures of both cell types (GBM + ECs) after 7 days of culture. Scale bars are 100 μm. (**c**) Corresponding image analysis of invasion frequency and distance for both singly and collectively invading GBM cells. ^◦◦◦◦^ Indicates P < 0.0001 when comparing mono- and co-cultures. (**d**) Confocal image analysis of nestin positive cells in GBM mono- and co-cultures after 3 and 7 days of culture and their respective invasion distances. ^◦◦◦◦^ Indicates P < 0.0001 when comparing time points of the same condition. * and **** Indicate P < 0.05 and 0.0001 when comparing mono and co-culture conditions. (**d**) Analysis of cellular nestin, SOX2, and Oct4 protein levels by flow cytometry of collagenase-digested mono- and co-cultures.

Perivascular niche conditions have been reported to guide GBM invasion and CSC maintenance^9^, and we observed that collagen invasion in our model system correlated with nestin positivity. Because of these connections we next tested the hypothesis that incorporation of brain endothelial cells into the 3D model promotes GBM invasion by elevating the number of stem-like cells. To test this hypothesis, mCherry-labeled human cerebral microvascular endothelial cells (hCMECs) were mixed into the collagen bulk prior to embedding CSC spheroids (Fig. 2a,b). Confocal image analysis of the co-cultures after 7 days indicated that the presence of hCMECs increased both the frequency and distance of GBM invasion with comparable effects on individual and collective cell migration (Fig. 2c). Importantly, co-culture with hCMECs largely prevented the decrease of nestin positive GBM cells in 3D monocultures (Fig. 2d, left). When co-cultured with hCMECs, nestin positive cells not only formed the majority of invasions, but they also migrated longer distances relative to nestin negative cells as well as relative to nestin positive cells in monocultures (Fig. 2d, right). These data suggest that hCMEC co-culture conditions stimulate invasion and that changes in stem-like behavior may have contributed. Indeed, flow cytometry analysis of nestin, SOX2, and Oct4 using cells isolated from the respective mono- and co-cultures by collagenase-digestion confirmed higher expression of stem cell markers in co-culture as compared to monoculture conditions (Fig. 2e). Interestingly, interactions between hCMECs and CSCs were bi- rather than uni-directional in this specific assay as confocal micrographs showed that the presence of GBM tumor cells promoted hCMEC alignment in the direction of CSCs invasion ultimately resulting in the co-localization of both cell types (Supplemental Fig. 1a,b).

### Endothelial cells stimulate GBM invasion in an IL-8 dependent manner

**Figure 3:**
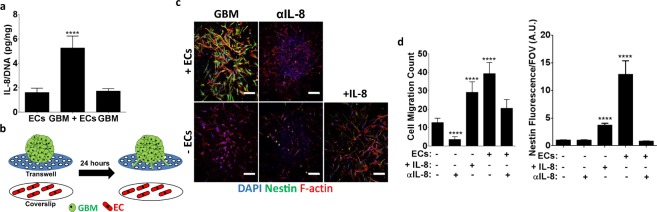
Interleukin-8 (IL-8) is increased in 3D endothelial cell-GBM co-cultures and modulates nestin levels and migration in a transwell assay. (**a**) IL-8 protein levels in collagen-embedded 3D mono- or co-cultures of endothelial cells (ECs) and GBM cells as measured by ELISA and normalized to fluorometrically determined DNA content. (**b**) Schematic of the transwell assay to test the effect of endothelial cell-secreted factors on GBM migration and nestin levels. (**c**) Confocal micrographs and (**d**) corresponding quantification of cell migration and nestin levels of migrated GBM cells on transwell membranes in the presence (+ECs) and absence (−ECs) of endothelial cells and with a neutralizing IL-8 antibody (αIL-8) or recombinant IL-8 (+IL-8). Scale bars are 100 μm. **** Indicate P-values < 0.0001 relative to spheroid monocultures with no endothelial cells, no IL-8 inhibitor, and no recombinant IL-8.

Given that hCMECs promoted GBM invasion and stem-like behavior in our co-culture model, we next sought to determine a potential molecular mechanism responsible for these observations. As IL-8 is secreted by endothelial cells, promotes stem-like behavior in GBM and other tumor cells^18,31^, and stimulates migration^18^, we hypothesized that endothelial cell-derived IL-8 may be a mediator of increased GBM tumor cell invasion in the 3D co-cultures. Interestingly, analysis of the 3D cell culture supernatant by ELISA indicated that IL-8 secretion per cell was significantly increased in co-culture conditions, as compared to monoculture conditions of either cell type (Fig. 3a). To determine more directly whether endothelial cell-secreted IL-8 alone could influence GBM migration and stem-like cell behavior, a transwell assay was conducted in which GBM spheroids were placed in the top chamber of the transwell and endothelial cell-seeded coverslips into the well below (Fig. 3b). Subsequently, the number of migrated GBM cells and their respective nestin levels were determined as a function of endothelial cell-derived IL-8 through adding an IL-8 function-blocking antibody to both monoculture and co-culture conditions or by supplementing recombinant IL-8 to GBM monocultures. Data from this analysis suggest that GBM tumor cells are capable of migrating through the membrane pores, but that brain endothelial cells not only enhance this effect, but also increase nestin levels in the migrated cells (Fig. 3c,d). Inhibiting IL-8 in the GBM monocultures decreased migration but had no effect on nestin levels. In contrast, inhibiting IL-8 in the co-cultures decreased both GBM cell migration and nestin levels. Consistent with these findings, supplementing GBM monocultures with IL-8 significantly increased migration as well as nestin levels (Fig. 3c,d). Collectively, these data suggest that IL-8 secreted by endothelial cells can drive GBM transwell migration possibly by stimulating GBM stem-like behavior.

**Figure 4:**
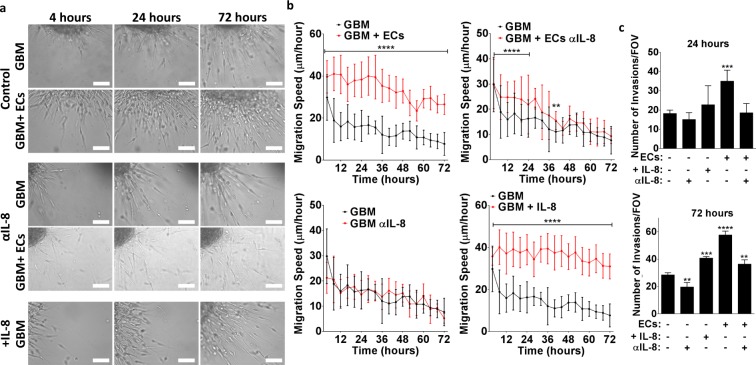
Endothelial cell-dependent IL-8-signaling promotes GBM tumor cell invasion into collagen at increased frequency and speed. (**a**) Representative bright field images of spheroid invasion over 72 hours collected by live cell imaging using a custom-made incubator microscope. Scale bars are 100 μm. (**b**) Corresponding image analysis of cell migration speed and (**c**) number of invasions as a function of co-culture with endothelial cells and/or IL-8 signaling. **, ***, and **** Indicate P-values < 0.01, 0.001, and 0.0001 relative to GBM cultures without endothelial cells, recombinant IL-8 and/or IL-8 antibody.

To confirm that endothelial cell-dependent stimulation of GBM invasion in 3D collagen gels was similarly dependent on IL-8, we next embedded GBM spheroids into collagen in the presence and absence of brain endothelial cells and stimulated or inhibited IL-8 as described above. Using time-lapse microscopy of these cultures for 72 hours combined with image analysis, we observed that GBM tumor cells invaded the collagen in all conditions, but that endothelial cells increased the number of invaded cells as a function of IL-8 (Fig. 4a–c). While GBM invasion was sustained over the 72-hour time frame, the speed at which GBM cells invaded decreased over time in conditions where endothelial cells were absent or where IL-8 signaling was inhibited (Fig. 4b). In contrast, supplementing IL-8 in GBM monocultures maintained invasion speeds at levels comparable to co-cultures with endothelial cells (Fig. 4b). Yet the number of cells that invaded in IL-8-stimulated GBM monocultures was lower relative to GBM co-culture with endothelial cells (Fig. 4c). Together, these data indicate that IL-8 constitutes an important signaling factor that promotes GBM tumor cell invasion into collagen in the presence of brain endothelial cells.

### Endothelial cell-stimulated GBM invasion correlates with an IL-8-dependent increase in nestin levels

**Figure 5:**
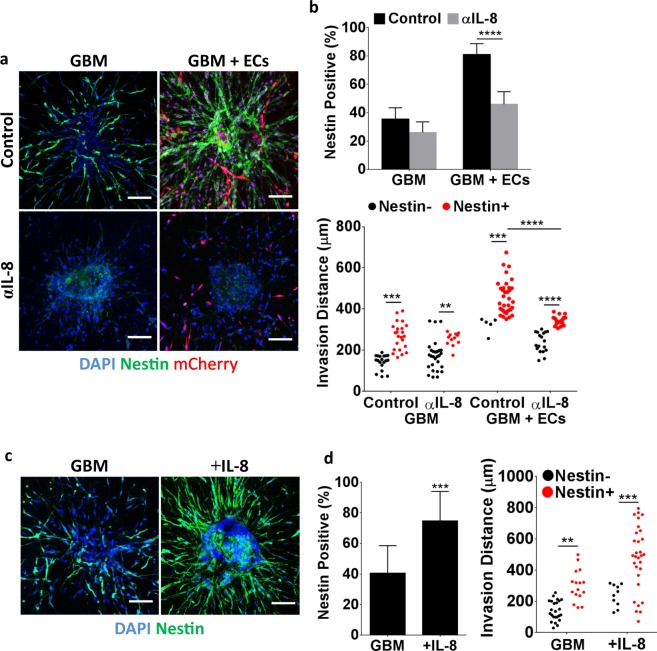
Endothelial cells increase GBM nestin levels in an IL-8-dependent manner. (**a**) Confocal micrographs and (**b**) corresponding image analysis of GBM nestin levels as well as invasion distances in the presence and absence of mCherry-labeled endothelial cells and with and without a function blocking IL-8 antibody (αIL-8). Scale bars are 100 μm. (**c**) Confocal micrographs and (**d**) corresponding image analysis of nestin-stained GBM monocultures with and without IL-8 supplementation. Scale bars are 100 μm. ** and *** Indicate P-values < 0.01 and 0.001.

To determine whether the detected increase in IL-8 mediated GBM invasion correlates with elevated nestin levels and thus, potentially stem-like behavior, we next determined nestin immunoreactivity in GBM mono- and co-cultures as a function of IL-8 signaling. Indeed, inhibiting IL-8 with a function-blocking antibody not only reduced GBM tumor cell invasion by 55%, but also significantly diminished the number of nestin positive cells in GBM-endothelial cell co-cultures (Fig. 5a,b). Accordingly, addition of recombinant IL-8 to GBM monocultures increased invasion by 82%, which correlated with a marked increase in the number of nestin positive GBM cells (Fig. 5c,d). Importantly, this IL-8-dependent increase in nestin-positive cells directly contributed to the endothelial cell-mediated increase in GBM tumor cell invasion as inhibiting IL-8 in the 3D co-cultures significantly reduced the number of nestin-positive cells that invaded far (>400 μm) into the surrounding collagen matrix (Fig. 5b, bottom). Inhibiting IL-8 had a less pronounced effect on the distance traveled by nestin positive cells in the GBM monocultures, although supplementation with IL-8 enabled nestin positive cells to migrate farther away from the spheroids as well (Fig. 5d, right).

**Figure 6:**
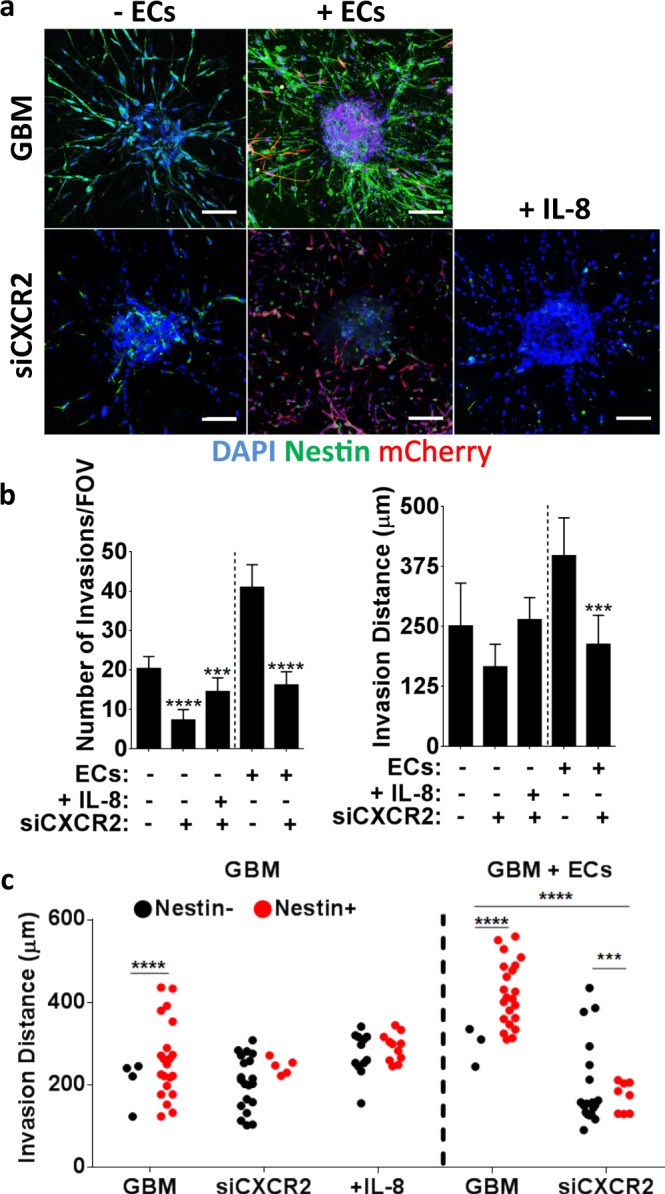
CXCR2 silencing reduces GBM spheroid invasion, which correlates with decreased nestin levels. (**a**) Confocal micrographs of nestin-stained GBM mono- or co-cultures prepared from tumor cells with siRNA-mediated reduction of CXCR2, and IL-8 supplementation. Scale bars are 100 μm. (**b**) Corresponding image analysis of spheroid invasion frequency and distance. (**c**) Invasion distances of nestin-negative and positive cells in mono-cultures of control or siCXCR2 GBM cells or in co-cultures with endothelial cells. ‘+IL-8’ indicates monocultures of siCXCR2 GBM cells with supplemented IL-8. *** and **** Indicate P-values < 0.001 and 0.0001, respectively.

Given that IL-8 regulates both GBM tumor cells and endothelial cells^18,22^, we cannot unequivocally conclude that the detected differences in GBM tumor cell invasion in our co-culture model were due to varied IL-8 signaling in the tumor cells themselves. To more directly determine the effect of IL-8 on GBM tumor cell invasion and nestin levels we repeated experiments using GBM tumor cells in which the IL-8 receptor CXCR2 was knocked down using siRNA as previously described and validated by our lab^18^. While IL-8 has two known cognate receptors, CXCR1 and CXCR2, our previous studies suggested that the knockdown of CXCR2 results in more pronounced reduction of the stimulatory effects of IL-8^18^. Accordingly, GBM spheroids formed by CXCR2 deficient cells exhibited significantly reduced nestin immunofluorescence and invasion relative to control spheroids (Fig. 6a,b). While IL-8 supplementation slightly stimulated invasion in CXCR2-knock-down spheroids, an effect that may be due to intact signaling via CXCR1, this treatment had no effect on nestin levels in siCXCR2-GBM cells (Fig. 6a–c). Accordingly, a comparison of nestin immunoreactivity with invasion distance suggests a requirement of CXCR2-dependent IL-8 signaling in promoting GBM invasion, with a more pronounced effect when GBM spheroids were co-cultured with endothelial cells (Fig. 6c).

### Inhibition of IL-8 signaling reduces spheroid size and tumor formation *in vivo*

**Figure 7:**
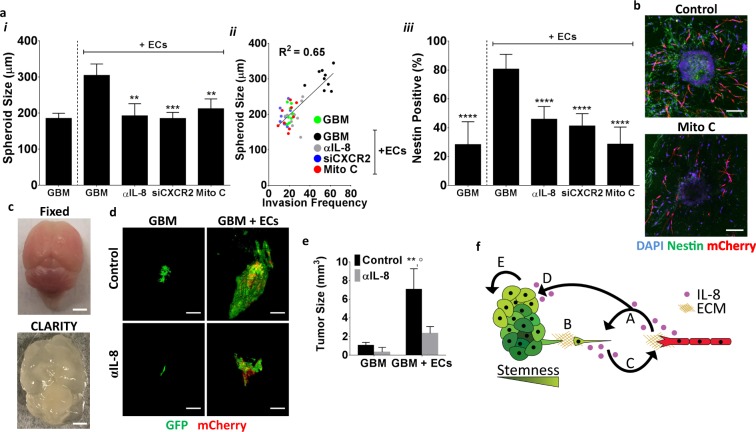
Endothelial cell-dependent GBM tumor growth and invasion are interdependent, and IL-8 plays a role in this process. (**a**) *i*, Effect of endothelial cells on GBM spheroid growth with (αIL-8, siCXCR2) and without blockade of IL-8 and with and without mitomycin (Mito C) treatment as determined by image analysis. *ii*, Correlation of spheroid size and invasion frequency. *iii*, Effect of endothelial cells on GBM cell nestin-positive frequency with (αIL-8, siCXCR2) and without blockade of IL-8 and with and without Mito C treatment. (**b**) Confocal micrographs of control and Mito C treated GBM co-culture spheroids. (**c**) Photographs of mouse brains prior to and after CLARITY treatment. Scale bars are 2.5 mm. (**d**) 3D reconstructions of light sheet micrographs visualizing tumor formation following intracranial injection of GFP-labeled human GBM tumor cells with and without mCherry-labeled hCMECs and with and without IL-8 antibody (αIL-8) treatment. Scale bars are 1 mm. (**e**) Corresponding quantification of tumor sizes. ** Indicates P < 0.01 when comparing mono- *vs*. co-injections; ^◦^ indicates P < 0.05 when comparing to IL-8 inhibition. (**f**) Schematic of the proposed mechanisms contributing to perivascular invasion of GBM. (A) IL-8 is secreted by endothelial cells in the perivascular niche of GBM. (B) Endothelial cell-secreted IL-8 induces GBM tumor cell migration and invasion of collagen-rich ECM. (C) Tumor cells secrete paracrine signals that induce endothelial cell migration and vascular sprouting towards GBM tumors. (D) IL-8 secreted by endothelial cells increases stem-like properties in GBM tumor cells that promotes malignancy by activating (E) GBM tumor growth and invasion.

Our *in vitro* results suggest that endothelial cells mediate GBM invasion in an IL-8-dependent manner, but to what extent these differences may be related to overall tumor progression remains unclear. Interestingly, we observed that co-culture with endothelial cells promoted not only invasion, but also spheroid growth. Image analysis of spheroid size revealed that the presence of endothelial cells increased the size of collagen-embedded GBM spheroids as a function of IL-8 signaling (Fig. 7a,i). Importantly, the endothelial cell- and IL-8-dependent increase in spheroid size positively correlated with invasion frequency (Fig. 7ai,ii). Blockade of either IL-8 with a neutralizing antibody or inhibition of cell proliferation with mitomycin C decreased both GBM spheroid size and invasion (Fig. 7ai,ii). These differences were likely related to an endothelial cell-dependent decrease in stem-like tumor cells as the percentage of nestin positive GBM cells and endothelial cell assembly into vascular structures decreased simultaneously with mitomycin treatment (Fig. 7a,ii,iii;b).

To determine the contributions of endothelial cells and IL-8 signaling on orthotopic GBM growth and invasion, immunocompromised mice were intracranially injected with either GBM tumor cells or a mixture of GBM cells and human-derived brain endothelial cells, and tumor formation was allowed to progress. During this period, GBM-implanted mice were subjected to weekly intraperitoneal injections of saline or the same function-blocking IL-8 antibody that was used for *in vitro* studies. To visualize the tumors, brains were made optically transparent by utilizing a CLARITY tissue clearing protocol to remove lipids from the tissue (Fig. 7c)^32^. Co-injection of GBM and endothelial cells increased tumor volumes ~4-fold relative to injection of GBM cells alone (Fig. 7d,e). Blocking IL-8 suppressed the growth-promoting effect of endothelial cells, while this treatment had only a negligible effect on tumor growth by GBM tumor cells alone. Importantly, co-injection of GBM and endothelial cells not only increased tumor sizes, but also led to less localized, more widely spread tumor formation mirroring our *in vitro* observations that IL-8 plays a role in endothelial cell-driven invasion of GBM tumor cells in the brain. Collectively, these observations suggest that brain endothelial cells stimulate GBM tumor growth in an IL-8 dependent manner and that this increase in growth possibly correlates with enhanced invasion.

## Discussion

Blood vessels are critical to GBM growth and invasion not only by supplying oxygen and nutrients, but also because endothelial cells are key to CSC self-renewal, proliferation, and migration^33^. However, the mechanisms by which the perivascular niche regulates CSCs and thus, invasion are not well understood. In this study, we utilized a collagen-based 3D culture model of GBM spheroids interacting with brain endothelial cells in combination with *in vivo* studies to analyze how interactions between both cell types modulate GBM invasion in the perivascular niche. Our results suggest that endothelial cells promote both GBM growth and invasion by stimulating the emergence of stem-like GBM cells in an IL-8-dependent manner (Fig. 7f) and that interference with this process may be explored therapeutically.

In the past, collagen type I has been identified as an important perivascular niche component that can directly increase CSC marker expression in GBM tumor cells^11^. However, these previous studies were performed in 2D culture, did not test GBM invasion, and lacked endothelial cells, which constitute a key cellular component of the perivascular niche that can independently promote GBM stem-like behavior^18^. To assess the potential functional contributions of these parameters, we embedded patient-derived GBM spheroids into collagen type I and found that collagen reduced rather than stimulated CSC marker expression under 3D culture conditions relative to suspension culture of GBM spheroids. Nevertheless, GBM tumor cells readily invaded into collagen, which positively correlated with an increase in stem-like tumor cell behavior. The presence of endothelial cells further enhanced 3D invasion of GBM tumor cells due in part to supporting stem-like behavior via paracrine functions (Fig. 7f). As GBM tumor cells reciprocally promoted hCMEC sprouting and alignment in the direction of CSCs invasion, the resulting co-localization of both cell types possibly contributes to perpetuating GBM invasion in the perivascular space (Fig. 7f). It has to be kept in mind that nestin is an intermediate filament protein that mediates its effect on tissue development and regeneration in part by regulating cytoskeletal dynamics^34^. In fact, nestin-dependent cytoskeletal changes likely contribute to the observed differences in CSC migration and proliferation potential in our studies^35^. While our study largely focused on the role of endothelial cell-derived paracrine signals in this process, direct cell-cell contact between both cell types likely plays a similarly important role^35^. Future studies will be needed to further examine this possibility.

IL-8 is a potent chemokine that is secreted by GBM and endothelial cells and can activate both stem cell- and angiogenesis-related signaling pathways^17,18,36^. However, which effect IL-8 has on GBM stemness and invasion in the context of the perivascular niche remains poorly understood as conventional culture models do not mimic cellular secretion of and responses to IL-8 in a pathologically relevant manner^24,25^. Using collagen-embedded spheroids of patient-derived GBM in combination with orthotopic xenografts, our results indicate that endothelial cell-dependent secretion of IL-8 increases GBM growth and invasion due in part to increasing the stem-like population of GBM tumor cells. While both IL-8 receptors, CXCR1 and CXCR2, can regulate tumor cell proliferation and metastasis in a variety of cancers, signaling via CXCR2 appears to have a more pronounced effect^16,37,38^. Accordingly, silencing of CXCR2 diminished endothelial cell-induced spheroid growth, stem cell marker expression and invasion in co-cultures, and also blocked these effects in monocultures that were supplemented with IL-8. These results support that IL-8 is a critical mediator of GBM invasion in the perivascular niche. The observation that silencing CXCR2 had a more pronounced effect than antibody-based blockade of IL-8 may be due to the promiscuity of CXCR2 as ligands other than IL-8 can stimulate signaling through this receptor^39^.

Hydrogel-based model systems to study the migratory and invasive behavior of GBM frequently focus on mimicking interactions with brain parenchymal ECM components such as hyaluronic acid (HA). However, transition of GBM tumor cells from HA to a collagen-rich, perivascular-like ECM increases invasion speed by modulating GBM cytoskeletal remodeling^40^. In fact, GBM tumor cells invading into collagen can promote their own invasion by clustering and aligning collagen fibers, which stimulates pro-invasive mechanosignaling via a positive feedback mechanism^41,42^. Consistent with these findings we observed that collagen-embedded GBM spheroids readily invaded into collagen. Nevertheless, over time the frequency of CSCs decreased. GBM CSCs have previously been reported to be largely insensitive to ECM stiffness^43^ and it is possible that increased ECM stiffness may down-regulate stem cell markers^44^. This could explain our finding that the frequency of nestin-positive cells decreased over time in collagen-embedded monocultures of GBM spheroids. Nevertheless, changes in collagen structure due in part to cell-mediated fiber alignment not only affect the tumor cells, but can also stimulate endothelial cell proliferation, secretion of IL-8, and consequential changes of vascular assembly^45,46^. Because of these connections and the importance of IL-8 in stimulating stem-like tumor cell behavior, we propose that endothelial cells present in the co-culture models assemble into vascular structures that maintain CSCs due in part to secreting enhanced levels of IL-8 (Fig. 7f). Consistent with an increase in CSCs in the co-cultures, GBM spheroids grew more and were more invasive in the presence of endothelial cells, and blocking IL-8 signaling in these cultures reduced these effects. Accordingly, endothelial cell-GBM crosstalk was critical for orthotopic tumor progression *in vivo* and depended on functional IL-8 signaling validating our *in vitro* results under more physiologic conditions. Nevertheless, it should be noted that the intraperitoneally injected IL-8 antibody probably elicited its effect in multiple ways: As the blood brain barrier (BBB) in GBM is leaky and dysfunctional, direct effects on the tumor cells are possible^47,48^. Nevertheless, normalization of stromal cell behavior including vascular functions likely played a role as well^49^. Indeed, IL-8 mRNA and secretion levels are increased in GBM and a variety of other cancers and are linked to increased therapeutic resistance suggesting that our findings may be of potential clinical significance^50^. Our analysis of gene expression data utilizing the National Cancer Institute (NCI) Glioblastoma Discovery Portal further indicates that increased IL-8 expression correlates with significantly reduced survival time in patients (Supplemental Fig. 2). Nevertheless, various other molecular mechanisms may have contributed to our findings and should be studied further. For example, matrix metalloproteinases (MMPs) are clinically associated with glioma progression and are influenced through IL-8 signaling^20,51^.

While our studies provide an increased understanding of how GBM-endothelial cell interactions in collagen-rich perivascular microenvironments contribute to the pathogenesis of brain cancer, these studies may be further advanced by considering the effect of additional ECM components, cell types, and mechanical inputs relevant to this disease. For example, HA is a key component of the brain ECM, HA receptors are markers for stemness and increased GBM tumor cell aggressiveness^52^, and HA-binding molecules such as osteopontin also increase aggressiveness^4,53^. Moreover, laminin is a main ECM component of the perivascular space and engagement of the laminin-binding integrin subunits α_6_ and α_7_ is critical to GBM CSC functionality as their inhibition attenuates CSC renewal and tumor formation^54^. Hence, developing model systems that include these components in addition to collagen would be of relevance^55,56^. Additionally, the brain perivascular niche contains cell types other than endothelial cells including pericytes and astrocytes that may independently modulate CSCs and invasion and hence should be considered in future studies^8,57^. As endothelial cell behavior and secretory profiles are altered under shear flow, integrating perfused microfluidic channels could help determine the relevance of these conditions on GBM invasion^58,59^. This approach has been used previously to study GBM-vascular interactions and could be expanded to determine their role in directing GBM invasion as a function of perturbed flow conditions and the resulting intracranial pressure^60^.

Our data provide evidence that brain endothelial cells promote GBM growth, stemness, and invasion and that these changes are linked to IL-8 signaling. These results are possibly applicable to other cancers where CSCs and IL-8 signaling have been shown to play a role in disease progression, such as in breast or prostate cancer. It will be of interest to determine whether or not the microvasculature plays a role in modulating disease progression in these patients as well^61,62^. Collectively, our findings underline the importance of relevant model systems to investigate tumorigenesis and invasion more broadly and define molecular mechanisms that may be explored therapeutically.

## Methods

### Cell culture

Patient-derived GBM tumor cells (provided by Dr. John Boockvar, Weill Cornell Medicine) were isolated as described previously^63^. Briefly, the lineage was generated from a GBM tumor resected from the left frontal lobe of a patient and whose cells exhibited a high mitogenic index and invasive profile alongside low p53 and high EGFR expression. This tumor also exhibited high vascular proliferation and necrosis upon post-surgical histological analysis. To enrich for CSCs, cells were suspended in media containing 1:1 of Dublecco’s Modified Eagle’s Medium and F12 supplement (Gibco) with added fibroblast growth factor (FGF) and epidermal growth factor (EGF) (20 ng/mL, Invitrogen) and 1% penicillin/streptomycin at 37 °C and 5% CO_2_. Cells were expanded in non-adherent culture flasks and media changed every 48 hours, with cells being centrifuged and re-suspended in fresh media. CSCs with silenced CXCR2 were generated by transducing cells with a lentivirus encoding CXCR2 shRNA as previously described and validated^18^. For experiments that required fluorescently labeled cells, CSCs were infected with MISSION® pLKO.1-puro-CMV-TurboGFP^TM^ positive control transduction particles according to manufacturer’s instructions (2 MOI, Sigma Aldrich) and were FACS-sorted using a FACSAria III (BD Biosciences) to select for highly green fluorescent cells.

Immortalized human cerebral microvascular endothelial cells (hCMECs) were generously provided by Dr. Babette Weksler (Weill Cornell Medical College, New York, NY)^64^. hCMECs were expanded with Endothelial Growth Media-2 (EGM-2, Lonza) at 37 °C, and 5% CO_2_ and used between passages p13 and 25 for experiments. Prior to experiments, mCherry-labeled hCMECs were generated by transfecting cells with a lentiviral vector encoding mCherry with help from the NYSTEM supported Cornell Stem Cell Modeling and Phenotyping Core. Labeled hCMECs were FACS-sorted using a FASCAria III to select for highly red fluorescent cells.

### Spheroid formation

GBM CSC spheroids were formed via aggregation on agarose-coated 96-well plates. Briefly, agarose (IBI Scientific, 0.015 mg/mL) was autoclaved and 50 μL of the warm solution was cast into each well. Subsequent cooling for 30 minutes at room temperature resulted in the formation of a concave surface to facilitate later cell aggregation. GBM cells were suspended at a concentration of 1,000 cells/mL in CSC culture media and 200 μL of this suspension was seeded into each well. The plate was then placed on an orbital shaker at low speeds and cells were allowed to aggregate for 3 days at 37 °C and 5% CO_2_. For spheroid size analysis, bright field images were acquired and the diameter of spheroids measured manually using ImageJ; for experimental use, GBM spheroids were gently flushed from wells using a micropipette and collected into microcentrifuge tubes for further use.

### Preparation of microfabricated hydrogel cultures

Poly(dimethylsiloxane) (PDMS, Dow Corning) was cast into 60 mm × 15 mm petri dishes and cut into strips measuring 45 mm × 20 mm × 15 mm. Subsequently, a biopsy punch was used to create five 6 mm holes into each PDMS strip (Acutech). PDMS strips were then plasma treated for 30 seconds and pressed onto plasma-treated glass cover slips (VWR). This allows for the activated surface of the PDMS and glass to bond, creating individual 15 mm-deep glass-bottom culture wells with liquid-proof seals. To prevent culture contraction at later stages of the protocol, the PDMS culture wells were further surface-treated to enable covalent linkage of collagen as previously described^65^. Prior to casting, a collagen I stock solution (Corning) was osmotically balanced with 10x concentrated EGM-2:DMEM:F12 (2:1:1), titrated with 1 N NaOH, and then diluted with the cell-media suspension (EGM-2:DMEM:F12 (2:1:1) to a final concentration of 0.6% (6 mg/mL) collagen. GBM spheroids were suspended in the collagen solution with or without hCMECs (0.5 * 10^6^ cells/mL) depending on experimental conditions. This suspension was cast into the PDMS culture wells to yield one spheroid per well and allowed to gel at increased temperature. All cultures were maintained for 7 days in EGM-2:DMEM:F12 (2:1:1) culture media that was replaced daily. To inhibit IL-8, a function-blocking antibody (HuMax IL-8, 20 μg/mL, Cormorant Pharmaceuticals) was added to culture media, while recombinant IL-8 (50 ng/mL, Genscript) was used for IL-8 supplementation. For quantification of IL-8 secretion, fresh media was added and collected after 24 hours of incubation. IL-8 concentration in the collected media was quantified using an enzyme-linked immunosorbent assay according to manufacturer’s instructions (ELISA, R&D Systems) and normalized to DNA content using a Quantifluor dsDNA system (Promega).

### Cryosectioning and immunostaining of spheroids

GBM spheroids were fixed in 4% paraformaldehyde (PFA), embedded in OCT (Tissue-Tek), and cyrosectioned to yield 20 μm-thick sections. For immunostaining, sections and cells were permeabilized and blocked using 0.5% Triton-X (VWR) and 2.5% Bovine Serum Albumin (BSA) followed by incubation with primary antibodies against SOX2 (1:200, R&D Systems), Oct4 (1:200, ABCAM), and nestin (1:100, Millipore). Secondary antibodies (1:500, anti-mouse conjugated Alexafluor 568, anti-goat conjugated Alexafluor 647, anti-rabbit conjugated Alexafluor 488, respectively) were diluted in PBS containing 4′,6-diamidino-2-phenylindole (DAPI, 1:5000) for nuclear counterstaining. Imaging was performed on a Zeiss LSM 710 confocal microscope.

### Immunofluorescence and fluorescence analysis of 3D hydrogel cultures

For hydrogel analysis, each sample was fixed in 4% PFA, permeabilized with 0.5% Triton-X and blocked with 2.5% BSA, and then stained against nestin (1:100, Millipore) overnight at 4 °C. After washing, samples were incubated with secondary antibody (1:500, anti-rabbit conjugated Alexafluor 488) mixed with DAPI (1:5000). Imaging was performed on a Zeiss LSM 711 or Zeiss 880 confocal microscope and z-stack images were collected in 4 μm step sizes up to 250 μm for each spheroid. Confocal image analysis of CSC and EC behavior was performed using ImageJ. Cell invasion frequency and distance were assessed using the spheroid periphery as the starting boundary. Individual GBM cells were defined as cells that were not in contact with other GBM cells and/or were physically connected to the spheroid. For spheroid size analysis, the long axis of spheroids was measured using the line measurement tool in ImageJ. Directional orientation of GBM cells and endothelial cells was measured using the angle parameter in ImageJ.

### Transwell migration assay

Transwell inserts (Corning Falcon HTS FluorobBlok Inserts, 8 μm pore size) were coated with 1% collagen I (Corning) and placed in 24-well plates. For conditions containing hCMECs, coverslips coated with 1% collagen I and seeded with 500,000 cells/mL were placed into the chamber below the transwell. Following 18 hours of migration, transwell membranes were removed, fixed with 4% PFA, permeabilized with 0.5% Triton-X, and then blocked with 2.5% BSA. To assess GBM migration through the transwell membrane, the underside of the transwell membrane was stained against nestin (1:100, Millipore), F-actin, and DAPI as described above. Subsequently, transwell membranes were mounted onto glass coverslips and their underside imaged at 200x magnification. 4 transwells per condition were analyzed.

### Flow cytometry

To quantify changes in stem cell marker expression, spheroids were analyzed using a BD Accuri (BD Biosciences) flow cytometer. To this end, spheroids were dissociated via gentle pipetting and cells were processed and measured using the Aldefluor assay (Stem Cell Technologies) in accordance with manufacturer’s instructions. Collagen-embedded spheroids were removed from the cultures through treatment with collagenase type 1 (1 mg/mL, Millipore Sigma) for 30 minutes, and dissociation as described above. Samples were fixed, permeabilized, and stained for SOX2, Oct4, and nestin and stained using the appropriate secondary antibody conjugated with Alexafluor 488. Analysis was conducted using the BD Accuri software to determine changes in stem cell marker expression as a function of fluorescence intensity.

### Time-lapse imaging

Time-lapse imaging was carried out using a miniaturized, lab-built microscope placed inside a conventional CO_2_ incubator. The instrument has bright field and fluorescence capabilities and consists of an ASI MS-4 XY stage, LED light sources, various magnification Olympus Objective lenses (4X Plan Achromat 0.10 NA, and 10X UPlanFLN 0.30 NA) and two CCD cameras from FLIR Systems Inc. (Chameleon CMLN-13S2M 1.3 MP Monochrome camera with a SONY ICX445 1296 × 964 pixel CCD). System software (Incuscope.exe) is written in Visual Basic under Microsoft Visual Studio 2013. Individual cells were tracked in 4-hour increments and the average migration frequency was binned into 24 hour increments while migration speed was averaged and recorded. All images were analyzed using ImageJ. To control for possible sample drift during imaging, reference points in each sample were selected, tracked over time, and used for correction.

### Animal studies and tumor imaging

All experimental protocols were approved by the Institutional Animal Care and Use Committee (IACUC) at Cornell University in accordance with all applicable federal, state and local regulations. All methods were carried out in accordance with approved guidelines. For tumor cell injections, 10-week old, male, NOD SCID mice were anesthetized with isoflurane at 1–2% oxygen levels, with body temperature maintained by a feedback-controlled heat pad at 37.5 °C. Atropine sulfate (0.05 mg/kg mouse weight) was injected subcutaneously (s.c.) to suppress lung secretions; s.c. injections of glucose (5% in saline, 100 µl/10 g/hour) were performed for additional fluid therapy. The skin was injected with bupivacaine (0.125%, 100 µL) at the injection site, retracted and a burr hole was drilled, 1.5 mm lateral and 1.5 mm posterior to bregma. Cells were injected using a Nanoject through a glass pipette that was inserted 1.4 mm at a 45-degree angle. After injection, the skin was closed with surgical adhesive. For IL-8 inhibition, mice were injected intraperitoneally with anti-IL8 (HuMax IL-8, Cormorant Pharmaceuticals; 40 mg/kg (antibody/mouse weight) or an equivalent volume of saline after 24 hours, a second dose of anti-IL8 (20 mg/kg) was administered 8 days after implantation. Fourteen days after tumor injections, mice were anesthetized deeply with isoflurane and euthanized using an overdose of pentobarbital (120 mg/kg). For brain clearing and imaging using the CLARITY method brains were extracted and immersed in acrylamide monomer solutions overnight at 4 °C^32^ Subsequently, acrylamide-based tissue-hydrogel hybrids were polymerized in a water bath at 37 °C for 4 hours and then cleared in sodium dodecyl sulfate (SBC) solution (1:1:3 SBC: boric acid: distilled water) for 4 weeks at 37 °C. Samples were incubated successively with 20% 2,2′-thiodiethanol (TDE) and 47% TDE for 1 hour each at 37 °C. Brain tissue surrounding the initial injection sites was dissected and embedded into 1.5% agarose mixed with 45% TDE prior to imaging. For light sheet imaging, tumor samples were submerged into a bath of 60% TDE and imaged on a LaVision Biotech light sheet microscope in 20 μm steps for the entire height of the sample. Arivis Vision4D software was used to render images and measure tumor size.

### Data mining

Data for GBM patient survivability as a function of IL-8 expression was gathered via the National Cancer Institute’s The Cancer Genome Atlas (TCGA) and the Glioblastoma Bio Discovery Portal. Data was sorted by longevity and z-score and the bottom and top quartiles were analyzed. Z-score measures were gathered from the database itself and represents the difference in standard deviation between tumor tissue and reference tissue.

### Statistical analysis

For all *in vitro* experiments at least 3 samples per condition were analyzed and experiments replicated 3 times unless otherwise indicated. Results are represented as the mean +/− standard deviation. All statistical tests were conducted in Graphpad PRISM utilizing a one- or two-way ANOVA with Tukey’s post-hoc test. P-values less than 0.05, 0.01, 0.001, and 0.0001 were considered statistically significant and were labeled with *, **, ***, or **** unless otherwise indicated.

## Supplementary information

Supplementary Data
