# Supplementary material for: Endothelial cells promote 3D invasion of GBM by IL-8-dependent induction of cancer stem cell properties
Source: Sci Rep. 2019 Jun 21;9:9069. doi: 10.1038/s41598-019-45535-y (PMC6588602; doi:10.1038/s41598-019-45535-y)

Endothelial cells promote 3D invasion of GBM by IL-8-dependent induction of cancer stem cell properties

Michael G. McCoy[1], Dennis Nyanyo[1], Carol K. Hung[1], Julian Palacios Goerger[1], Warren Zipfel[1], Rebecca M. Williams[1], Nozomi Nishimura[1], Claudia Fischbach[1,2]*

[1]Nancy E. and Peter C. Meinig School of Biomedical Engineering, Cornell University, Ithaca, NY 14853, United States

[2]Kavli Institute at Cornell for Nanoscale Science, Cornell University, Ithaca, NY 14853, United States

*Corresponding Author

Email: CF99@cornell.edu

Keywords: Glioblastoma, Cancer Stem Cell, Interleukin-8, Tumor Cell Invasion, Perivascular Niche

Supplemental Figure 1

(a) Confocal micrographs of the periphery of GBM co-culture spheroids (nestin, labeled green) visualizing directional alignment of endothelial cell (mCherry, labeled red) towards the spheroid. (b) Left: Quantification of the frequency of endothelial cell co-localization with GBM invasions ('sprouts') following 3 and 7 days of culture. Middle, Right: Measurement of the incident angle of both tumor cell invasions and endothelial cells relative to the spheroid surface demonstrate different growth and migration angles after 3, but not 7 days of culture suggesting increasing alignment over time. ** and **** indicate P-values less than 0.01 and 0.001, respectively.

Supplemental Figure 2

Analyzing data from The Cancer Genome Atlas (TCGA) with the National Cancer Institute's Glioblastoma Bio Discovery Portal suggested a negative correlation between IL-8 and patient survival by revealing that IL-8 z-scores were increased in patients with lower *vs.* upper quartile survival. LQ refers to lower quartile and UQ refers to upper quartile. * indicates a P-value of less than 0.05.

Supplementary Figure 1

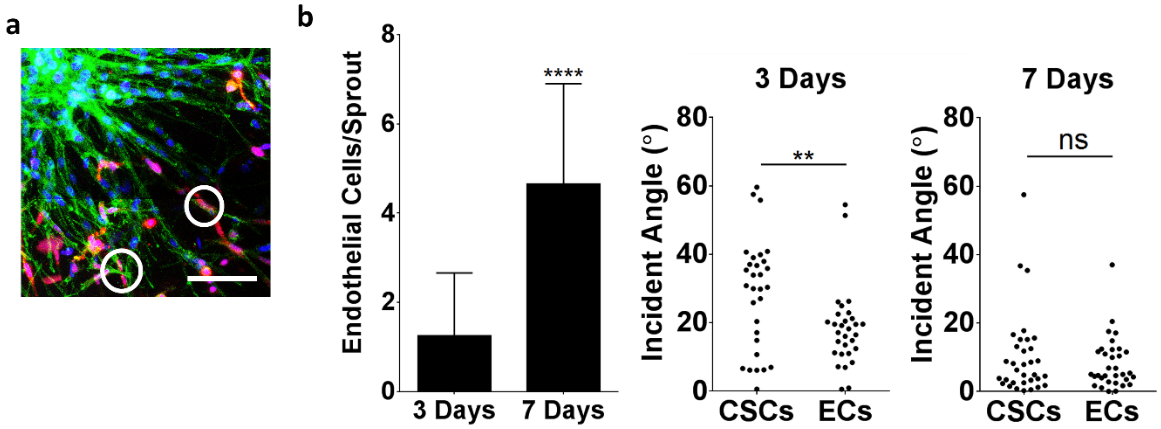

Supplementary Figure 2

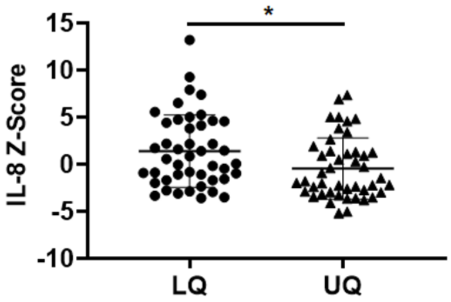

Supplement: Supplementary file 1 — Supplementary Data [file 41598_2019_45535_MOESM1_ESM.pdf]
